# Electrospun Polylactic Acid (PLLA) Microtube Array Membrane (MTAM)—An Advanced Substrate for Anticancer Drug Screening

**DOI:** 10.3390/ma12040569

**Published:** 2019-02-14

**Authors:** Chia-Hsuan Tseng, Wan-Ting Huang, Chee Ho Chew, Jun-Kai Lai, Shih-Hsin Tu, Po-Li Wei, Kang-Yun Lee, Gi-Ming Lai, Chien-Chung Chen

**Affiliations:** 1 Graduate Institute of Biomedical Materials & Tissue Engineering, Taipei Medical University, Xinyi District, Taipei 11031, Taiwan; vickyt250059@live.com (C.-H.T.); sandyhuang@mtamtech.com (W.-T.H.); chchew88@gmail.com (C.H.C.); 2 MTAMTech corporation, 17th floor, 3rd Yuanqu Street, Nangang District, Taipei 11503, Taiwan; dgenkai1109@gmail.com; 3 Department of Surgery, Taipei Medical University Hospital, Xinyi District, Taipei 11031, Taiwan; drtu@h.tmu.edu.tw (S.-H.T.); poliwei@tmu.edu.tw (P.-L.W.); 4 Division of Pulmonary Medicine, Department of Internal Medicine, Shuang Ho Hospital, Taipei Medical University, Taipei 235, Taiwan; leekangyun@tmu.edu.tw; 5 Division of Thoracic Medicine, School of Medicine, College of Medicine, Taipei Medical University, Taipei 250, Taiwan; 6 International PhD Program for Cell Therapy and Regeneration Medicine, College of Medicine, Taipei Medical University, Taipei 250, Taiwan; 7 Department of Internal Medicine, School of Medicine, College of Medicine, Taipei Medical University, Taipei 250, Taiwan; gminlai@tmu.edu.tw; 8 Ph.D Program in Biotechnology Research and Development, College of Pharmacy, Taipei Medical University, Taipei 250, Taiwan

**Keywords:** personalized medicine, anticancer drug screening, microtube array membrane (MTAM), hollow fiber assay (HFA), electrospinning

## Abstract

The advent of personalized cancer treatment resulted in the shift from the administration of cytotoxic drugs with broad activity spectrum to a targeted tumor-specific therapy. Aligned to this development, the focus of this study revolved around the application of our novel and patented microtube array membrane (MTAM) in the US National Cancer Institute (NCI) developed an HFA (hollow fiber assay) assay; hereinafter known as MTAM/HFA. Electrospun poly-L-lactic acid (PLLA) MTAM was sterilized and loaded with cell lines/patient derived tumor cells (PDTC) and subcutaneously implanted into the backs of BALB/C mice. Anticancer drugs were administered at the respective time points and the respective MTAMs were retrieved and the viability tumor cells within were quantified with the MTT assay. Results revealed that the MTAMs were excellent culture substrate for various cancer cell lines and PDTCs (patient derived tumor cells). Compared to traditional HFA systems that utilize traditional hollow fibers, MTAM/HFA revealed superior drug sensitivity for a wide range of anticancer drug classes. Additionally, the duration for each test was <14 days; all this while capable of producing similar trend outcome to the current gold-standard xenograft models. These benefits were observed in both the in vitro and in vivo stages, making it a highly practical phenotypic-based solution that could potentially be applied in personalized medicine.

## 1. Introduction

The development of anticancer drug research are often plagued with poor translatability between the developmental phases and the clinical phases [1]. This is often caused by the use of cancer cell lines in the research and development, where there are significant differences in terms of molecular markers between cell lines and primary tumors. In addition, the lack of heterogeneity in induced tumor models employed in the research and development phases further aggravates the poor translatability of outcome between various development phases [2,3]. Therefore, there is an urgent need for better models in anticancer drug research and development.

Patient-derived xenografts (PDXs), established from tumor tissue directly engrafted into immune-deficient mice, are one possible solution. There is increasing evidence that suggest PDXs consistently conserve biological features of the parental malignancies (histologic architecture, gene-expression, mutational status and metastatic potential), while maintaining the complex interaction between implanted tumor cells and its microenvironments [4,5,6,7,8]. Various research have demonstrated that there are correlations between PDX models and the corresponding clinical outcomes [9,10,11,12]; with growing number of research groups advocating the integration PDX models into current treatment of cancer patients in predicting clinical response [13,14]. In line with this development, large scale in vivo anticancer drug screening in 60 treatment regimens against a comprehensive collection of PDX models (1075 models across 15 cancer types) have successfully duplicated treatment response of previous clinical trials [15]. In addition, extensive analysis on a larger and more heterogeneous patient population has also revealed similar degree of accuracy [16]. In view of these outcomes, strong emphasis has been placed in the development of PDX models in anticancer drug screening.

About 20 years ago, Dr. Hollingshead, M. et al. at the National Cancer Institute (NCI)/NIH, USA, proposed a unique in vivo hollow fiber assay (HFA) that can dramatically reduce the screening time required, the number of animals and the quantity of required candidate compound [16,17]. This technology involves the encapsulation of cancer cell lines within the commercially available hollow fibers (HFs), which were then subcutaneously (SC) or intraperitoneal (IP) implanted into the respective animals, and followed by the administration of the candidate drug(s). At predetermined time points, the HFs were extracted and the cancer cell lines within the HFs quantified. Unlike traditional xenografts, the HFA screening of anticancer drugs only require two weeks as opposed to 60–90 days. Furthermore, a single animal can be implanted with multiple cancer cell lines (in different HFs) thereby resulting in a rapid, economical and significant improvement in the ethical use of animals in the experiments [18,19]. Despite these benefits, the HFA technique is plagued with one key weakness namely, the poor sensitivity towards anticancer drugs, which is the result of the thick lumen walls of hollow fibers (100–200 μm).

Recently, we developed a new class of hollow fibers known as the microtube array membrane (MTAM), which consisted of ultra-thin, homogenously porous, one-to-one connected individual fibers [20,21,22]. The unique microstructures conferred various benefits in various applications from ethanol fermentation, nerve regeneration and cancer studies [23,24,25,26,27,28]. By replacing the traditional hollow fibers used in the HFA process with our MTAMs, we aim to demonstrate the use of our MTAM-based HFA (MTAM/HFA) in personalized medicine. The use of conventional PDX models for personalized medicine are often plagued with weaknesses such as requiring a long testing time and large sample required for successful engraftment, which may not be available [29,30].

In this study, we strive to demonstrate the MTAM/HFA in screening of several types of cancer cell lines against several types of anticancer drugs in both in vitro and in vivo settings. In addition, we also will demonstrate the use of MTAM/HFA in the determination of the drug sensitivity of patient’s tumor cells, which could potentially increase the accuracy and sensitivity of anticancer drug screening within a clinically practical time frame.

## 2. Materials and Methods

### 2.1. MTAM Preparation

Preparation of poly-L-lactic acid (PLLA) microtube array membranes (MTAM) with different tube wall porosity has been reported previously [9,10]. Briefly, PLLA (BioTechOne, Taipei, Taiwan) and polyethylene glycol (Sigma-Aldrich, St. Louis, MO, USA) were dissolved in a cosolvent N,N-dimethyl formamide (DMF; Tedia, Fairfield, OH, USA) and dichloromethane (DCM; Mallinckrodt, St. Louis, MO, USA) to form a solution containing PLLA to PEG (polyethylene glycol) at a ratio of 7:3. The resulting solution was electrospun at a voltage of 5–7 kV provided by an electrostatic charger (You-Shang Co., Fongshan City, Taiwan) under ambient conditions (relative humidity of 50% + 5% and a temperature of 25 °C + 1 °C). The resulting MTAM were washed with double distilled water (ddH_2_O) and air dried for 24 h.

### 2.2. Cell Culture

Human bone chondrosarcoma, fibroblast-like (SW1353) cell line, fibro sarcoma (HT1080) cell line, human lung adenocarcinoma (A549), colon cancer cell line (HCT116) and glioblastoma multiform (GBM) were used and maintained in conditional medium. The cell culture and all assays in this study were performed in DMEM/F-12, RPMI, and HDMEM (GIBCO, Gaithersburg, MD, USA) supplemented with 10% FBS (Biological Industries, Kibbutz Beit-Haemek, Israel), 50 U/mL penicillin, and 50 mg/mL streptomycin (full medium, Biological Industries, Israel) at 37 °C in a 5% carbon dioxide atmosphere.

### 2.3. Cell Proliferation Assay

The respective fibers were washed three times using ddH_2_O and placed in a container containing ddH_2_O for ultra violet (UV) light sterilization. After sterilization, the fibers were flushed under sterile conditions. SW1353, HT1080, and A549 cells (2 × 10^5^ cells/10 μL) were drawn into a 0.5 cm × 1.5 cm PLLA/30 MTAM via capillary action; and into CellMax fibers measuring 1 cm. The ends of the respective fibers were sealed by pliers at 0.5 cm intervals several times. The cell containing fibers were then cultured in six-well plates filled with complete growth culture medium.

### 2.4. Cytotoxicity Study

The respective cells that were cultured within six-well plates were siphoned into the respective fibers at a density of 2 × 10^5^ cells/10 μL, and the ends of the fibers were sealed with a plier. Next, these fibers containing cells were maintained in six-well plates in the complete growth culture medium. The corresponding anticancer drugs were administered accordingly and incubated in 37 °C in a 5% CO_2_ atmosphere. At a predetermined time, fibers were cut into pieces then analyzed by the BCA protein assay kit (Pierce). The absorbance at 562 nm was measured by ELISA (Multiskan GO, Thermo Scientific, Waltham, MA, USA). All experiments were performed three times with 12 replicated wells for each sample and control per assay.

### 2.5. Preparation and Implantation of Cell-Loaded MTAM

All animal studies were approved by the animal research ethics committee (LAC-2016-0207). Thirty Male BALB/c mice at 6–7 weeks of age were purchased from BioLASCO Taiwan Co., Ltd. (Taipei, Taiwan) and were free of known pathogens at the time of use. All procedures were performed in compliance with the guidelines of the Institutional Animal Care and Use Committee, which are based on the guidelines of the Association for Assessment and Accreditation of Laboratory Animal Care including facility (protocol number LAc-2U14-U193). Mice were housed in the TMU Laboratory Animal Center (Taipei, Taiwan) in a conventional environment at constant temperature (20 °C ± 3 °C) and humidity (50% ± 20%). To prepare samples, a sterile field was established in a biological safety cabinet. Immediately prior to cell siphoning, the respective fibers were individually rinsed with condition media. The cell suspension (2 × 10^5^ cells/10 μL) was siphoned into a 0.5 cm × 1.5 cm PLLA/30 MTAM and CellMax fibers measuring 1 cm via capillary action. Next, the ends of the fibers were sealed by plier at 0.5 cm intervals several times until the ends of the fibers were not be cocked. After preparation, the samples were transferred to culture dishes containing complete medium and incubated overnight at 37 °C in a 5% CO_2_ atmosphere prior to implantation into mice. Anesthesia (methoxyflurane [Pitman-Moore, Inc., Mundelein, IL]) was induced into the respective mice by via inhalation. For subcutaneous (s.c.) implants, a small skin incision measuring 1–2 cm was made at the nape on the back. Samples were inserted with a caudal through the subcutaneous tissues. The skin incision was closed with a skin staple. After 24 h of cell proliferation, the animals were treated with the corresponding anticancer drugs. The respective fibers were retrieved at the respective predetermined time points. A general clinical observation (e.g., in life numbers, body weight, food consumption) was made twice daily by a veterinarian.

### 2.6. Patients

This study included two patients with colorectal cancer (IRB: N201512059), heck and neck cancer (IRB: N201704053) and breast cancer (IRB: N201604012) whom were diagnosed at Taipei Medical University Hospital (TMUH). Surgical specimens and core needle biopsy were taken before any treatment. All patients signed an informed consent for the usage of their tumor samples for research.

### 2.7. Response Evaluation Criteria In Solid Tumors (RECIST)

Responses to therapy were then converted to clinical outcomes (based on RECIST) from changes in tumor volume over the course of treatment.

### 2.8. Statistics

Data are presented as mean values ± SD. Statistical significance was analyzed by two-tailed Student’s t-test. Values of P < 0.05 (n = 5) were considered significant.

## 3. Results

### 3.1. Cell Culture of Cancer Cell Lines in PLLA MTAM

**Figure 1:**
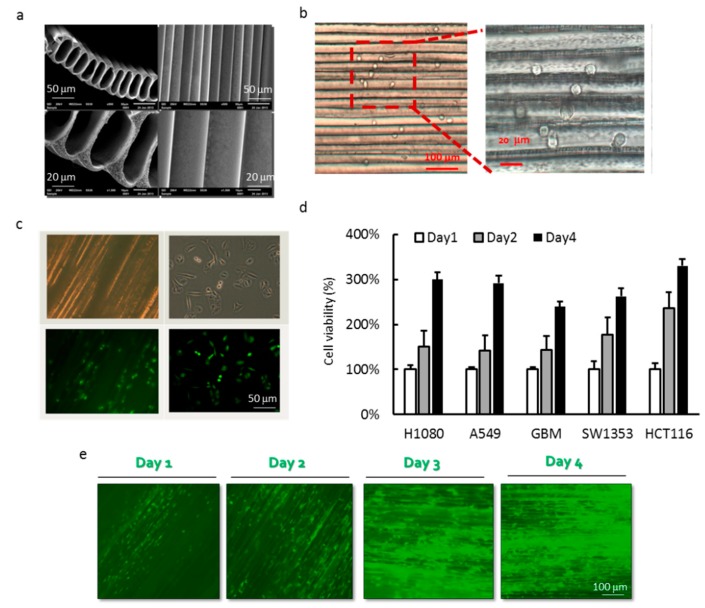
(**a**) SEM view of the top and transverse section of the MTAM depicting the microstructure of the electrospun MTAM; (**b**) optical microscopy image of the A549 cells loaded (siphoned) into the respective lumens of the MTAM; (**c**) the GFP-GBM cells that were loaded at a cell density of 1 × 10^6^ cells/mL and its corresponding optical microscopy image; (**d**) cell viability of various cancer cell lines that were cultured in MTAM that demonstrated excellent cell viability; and (**e**) Calcien-AM stained optical microscopy images of GBM cultured on MTAM. Results are expressed as mean data from six fibers (Student’s t-test; P < 0.05).

The PLLA MTAM appeared to be an off-white thin film to the naked eye. Conversely, when observed under the SEM (TM3030, Hitachi, Tokyo, Japan), the unique microstructures consisted of one-to-one, individually connected submicron scale hollow fibers. The surface of the respective MTAM consisted of homogenously distributed pores of about 200 nm. In terms of the dimensions of the individual lumens, the height × width measured 60 μm × 40 μm, and a lumen wall thickness of 2.2 ± 0.4 μm (Figure 1a). The PLLA MTAM appeared transparent under the optical microscope and the cells cultivated on the inner lumen surface appeared to be attached to it (Figure 1b). GF-GBM cells were cultured for 24 h in both the PLLA MTAM and culture dish, followed by fluoroscopy examination of the morphology. The morphology of GBM appeared to closer to the morphology found in in vivo settings as opposed to those observed in culture dishes (Figure 1c). Five types of cancer cells; A549, SW1353, HT1080, GBM and HCT116 were cultured within the MTAM in an in vitro setting and all of the cancer cells lines proliferated well in MTAM (Figure 1c). In the case of GBM, the green fluorescent gene transfected GBM cell directly showed the growth of cell in MTAM under the observation of florescent microscopy. The results demonstrate that our novel hollow fiber tube can be used to culture cancer cell lines.

### 3.2. Effects of MTAM/HFA System on the Cytotoxicity of Cancer Cells

**Figure 2:**
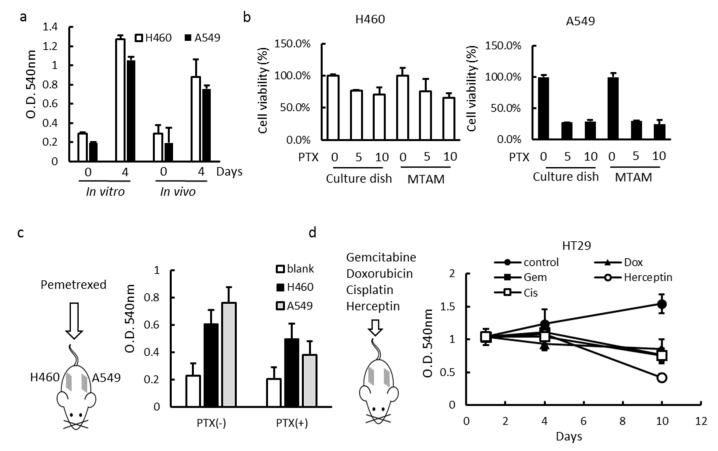
Anticancer drug screening of the MTAM-based HFA system (**a**) in vitro and in vivo cell culture of H460 and A549 which demonstrated superior cell proliferation within 4 days when cultured using MTAM as a substrate, the growth inhibition of H460 and A549 cells by pemetrexed (**b**) in vitro or (**c**) in vivo. (**d**) Growth inhibition of HT29 cells in the MTAM implanted s.c. in mice. Drugs were administrated once daily by i.p. injection from days 3–7 after implantation (Student’s t-test; P < 0.05).

The need for characterizing the initial cell loading density of hollow fibers for improving compound efficacy has been shown earlier [31]. During the treatment period cytotoxicity was assessed using the “stable end point” modified MTT ((3-(4,5-Dimethylthiazol-2-yl)-2,5-Diphenyltetrazolium Bromide) assay. First, H460 and A549 cells seeded at 2 × 10^5^ cells/10 μL were defined as the optimal seeding density for in vitro and in vivo growth within hollow fibers over 4 days (Figure 2a). Additional in vitro and in vivo MTAM/HFA studies involving pemetrexed treatment were performed using this cell seeding density. When compared the cell culture dish, the drug sensitivity in these two conditions were similar in this two cell lines (H460 ≤ 50%, A549 ≥ 50%) (Figure 2b). Next, we tested the cell toxicity in an in vivo setting. The MTAM contained individual cancer cell was subcutaneous transplanted onto two different sites, located on the back of a single mice; which was followed by the standard pemetrexed (100 mg/kg; i.p.) treatment. At day 10, fibers were excised, and the cell viability results revealed cells retrieved from fibers from pemetrexed-treated mice to have the same drug sensitivity (when compared to in vitro data) in these two cells (Figure 2c). We subcutaneously transplanted an empty MTAM as a blank to avoid contamination from host cell. On the other hand, we used 15 mice divided into five experimental groups: control, Gemcitabine, Doxorubicin, Cisplatin, and Herceptin. The fibers were excised from the mice at day 4 and 10. Compared to the control groups, Herceptin treatment produced a significant reduction in net growth of about 50% for HT29 (P < 0.01) (Figure 2d). Taken together, these results proved that our MTAM/HFA can be applied for multi-drug and multi-cancer cells conditions screening in a one-time operation.

### 3.3. In Vitro and In Vivo Studies of Drug Sensitivity of Cancer Cell Lines Cultured within MTAM/HFA and PVDF(Polyvinylidene Fluoride) HFA

**Figure 3:**
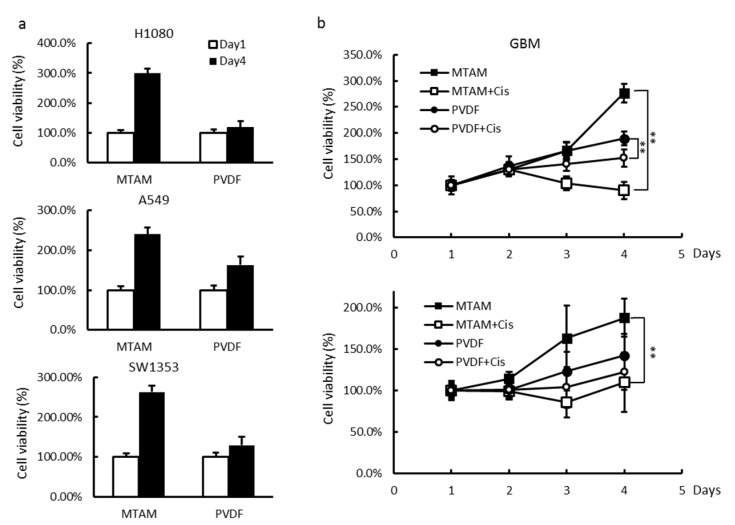
Comparison of cell growth rate and drug sensitivity between MTAM and PVDF HF. (**a**) H1080, A549 and SW1353 cells loading in MTAM or PVDF-HF at 1 × 10^6^ cells/mL, were incubated at 37 °C and retrieved at each time point. (**b**) Growth inhibition of GBM cells in the MTAM or PVDF-HF in growth medium (top right) or implanted s.c. in mice (bottom right). Drugs were administrated once daily by i.v. injection from days 3–7 after implantation. Student’s t-test; P < 0.05.

In the traditional HFA system, PVDF hollow fibers were employed and in this study served as a benchmarked. Comparatively, the cell count within the PLLA MTAM was one to two orders higher than the cell count within PVDF HF systems (Figure 3a). Upon administration of Cisplatin, there was higher rate of decrease of cancer cells within PLLA MTAM than that of PVDF systems (Figure 3b). Cell count within PLLA MTAM appeared to be suppressed at a low concentration of 15 μM as oppose to 50 M for the cell count within the PVDF systems (data not shown). Regardless of dosage, Cell toxicity sensitivity of Cisplatin was observed to be higher in MTAM-based systems. Similar outcomes can be observed in the in vivo cell toxicity test. Furthermore, the MTAM system registered a significantly higher difference in terms of cell viability between the positive and negative control in the in vivo setting. The corresponding CellMax system barely registered any difference in cell viability between the positive and negative control (Figure 3b). Comparatively, cancer cells cultured within MTAM appeared to be sensitive to cisplatin even at a relatively concentration of 15 μM as oppose to cancer cells cultured within CellMax which exhibited very little reaction to cisplatin at low concentration.

### 3.4. Comparison of MTAM vs. PDX as a Primary Culture Substrate

**Figure 4:**
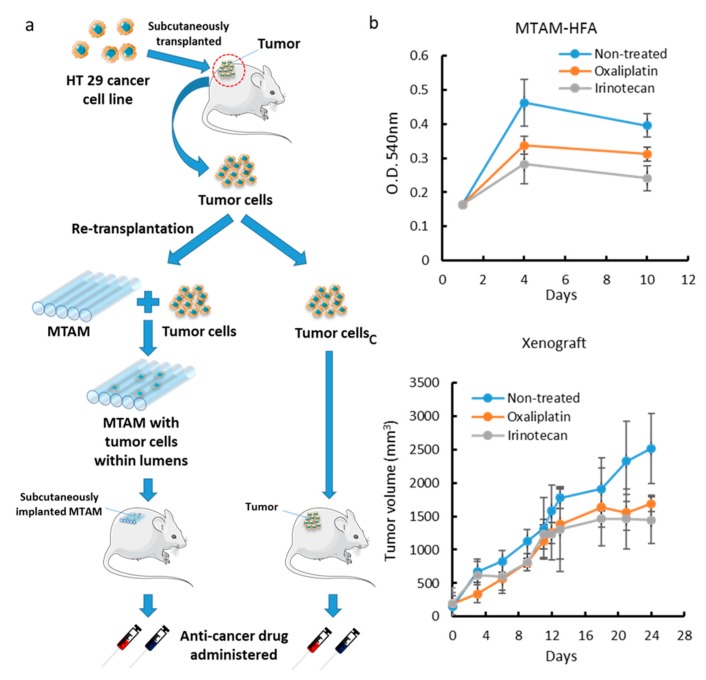
The Mouse PDX model was used to evaluate the reliability of MTAM PDX system (**a**) HT29 cancer cell line were injected into mouse model to establish a tumor, and this mice is treated as a ‘patient’. The resulting tumor were extracted and divided into two, where one part was utilized in the MTAM-based HFA system and the other in a standard xenograft model to serve as a benchmark. (**b**) Growth inhibition of primary HT29 cells in the MTAM implanted s.c. in mice. Drugs were administrated once daily by i.v. injection from days 3–7 after implantation. (**c**) Nude mice bearing 100 mm^3^ xenografts were randomized into treatment groups, Tumor volume of mice bearing primary HT29 xenografts was measured every other day. Both (**b**) and (**c**) appeared to provide similar screening outcome trend and to a certain extent comparable outcome, which suggested that the MTAM PDX system was capable of producing similar outcome when compared to the current gold standard (traditional xenograft) Student’s t-test; P < 0.05.

In order to develop the application of MTAM for clinical usage, we simulated the clinical situation and tried to establish a personalized anticancer drugs selection platform for clinical patients. Here, we first created a tumor specimen by colon cancer cell line HT29 by subcutaneous injection in nude mice. A few days later, the tumor was removed from the mouse and divided into two parts: one for MTAM, another was re-subcutaneously transplanted into a new nude mouse. Both groups were grown for an appropriate period of time for colorectal cancer drug testing (Figure 4a). The two models assessed in our analysis were treated with two cancer drugs: Oxaliplatin and irenotecon, and cell proliferation was measured by MTT assay or tumor volume. The results were shown in Figure 4b,c. We found the MTAM group showed more sensitivity to irinotecan. Similarly, the xenograft group was also more responsive to irinotecan. It’s worth noting that the MTAM group required less time than the xenograft group by a significant margin. Thus, the results suggested that the MTAM system can be a highly rapid alternative screening system to xenograft models which could assist many oncologists in determining the best anticancer drug for a particular patient within a clinically practical timeframe.

### 3.5. MTAM/HFA as a Potential Platform for Anticancer Drug Screening in Personalized Medicine

**Figure 5:**
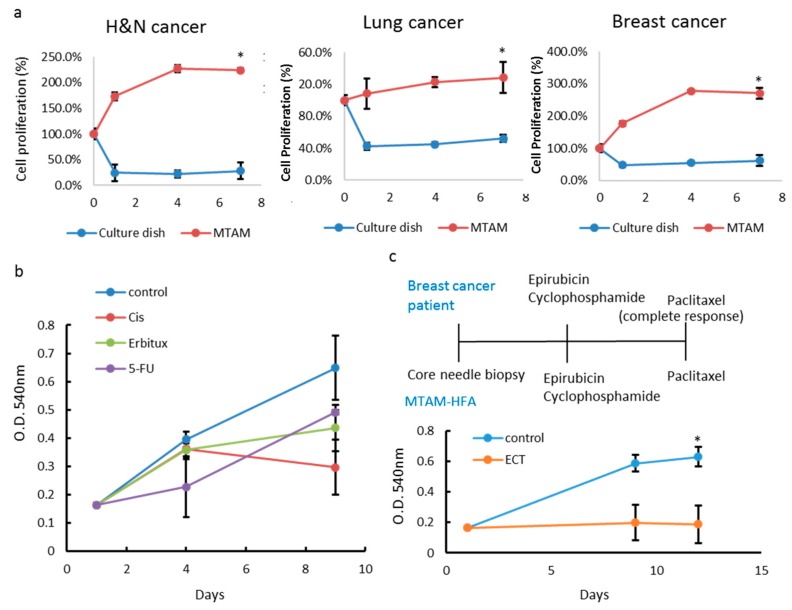
PDTC models accurately replicate patient responses. (**a**) Graph depicting different tumor types growth in culture dish or MTAM to establish PDTC. (**b**) PDTC colon cancer models were screened by different type of anticancer drugs. (**c**) PDTC models were screened against the corresponding therapies received by the patient. Graphs show the average implanted cell viability for three to nine animals and SD. * Treated groups significantly different from untreated controls at the end point of the experiment (Student’s t-test; P < 0.05).

For establishment of MTAM-based PDTC for drug selection, we divided digested-tumor specimen from three patients in three different tumor types into two conditions. One was loaded into MTAM then cultured in optimal medium, another was directly seeded. Tumor samples were obtained from core biopsies (breast cancer; head and neck cancer), or surgical excisions (colon cancer). The cell viabilities from these two groups were detected by MTT assay. The results demonstrated that patient derived tumor cells had better proliferation rate in MTAM (Figure 5a). Then, we tested the drug sensitivity of colon cancer PDTC in vivo. The MTAM containing patient’s cancer cell was subcutaneous transplanted in BALB/c mice and the fibers were excised from the mice at day 4 and 10. Compared to the control groups, Cisplatin treatment produced a significant reduction in net growth of about 50% in this case (P < 0.01) (Figure 5b). Taken together, these results proved that our MTAM-based PDTC (MTAM/HFA-PDTC) can be applied in anticancer drug selection. We also tested some of MTAM/HFA-PDTCs against therapies that yielded a positive outcome in the corresponding patient (Figure 5c). MTAM/HFA-PDTCs were tested against the same treatment received by the patient, and responses assessed using changes in cell viability. A positive response was designated as positive and correlative to clinical outcome of patients if a RECIST equivalent of complete response (CR), partial response (PR), or stable disease (SD) was observed. As an example, Figure 5c shows the PDTC results for a female with stage III breast cancer. This patient will take the Neoadjuvant chemotherapy. After treatment with the same regimen, tumor cell cannot even grow within MTAM.

## 4. Discussion

The nanoporous PLLA MTAM possessed self-supporting structure can be easily handled and manipulated into most the configurations as desired throughout the experimental procedures. In regards to the cell loading procedure, the process primarily employed the capillary force that existed in the respective lumens, thereby siphoning the droplet of cell culture media up into these lumens. Additionally, due to the transparent wall of the PLLA MTAM, the tumor cells that were siphoned into the PLLA MTAM were clearly visible. It was observed that the PLLA MTAMs were not as mechanically sound as CellMax fibers; nevertheless, this did not hamper the use of the PLLA MTAMs throughout the duration of the experiment as the PLLA MTAMs were sufficiently strong. For ease of tracking, the PLLA MTAM can be colored through the addition of food grade dye to the polymer solution prior to electrospinning. The addition provided a high contrast between the PLLA MTAM and the surrounding soft tissue; a highly beneficial characteristic for ease of identification of the PLLA MTAM.

Results indicated that the MTAM with the diameter size between 50 and 60 μm performed the best in terms of cell survivability. Potentially, at this diameter, oxygen was allowed to freely permeate deep into the respective PLLA MTAM thereby allowing tumor cells in the PLLA MTAM to survive better since oxygen appeared to be one of the most important nutrient for cells proliferation [32]. An alternative explanation could be because of cells aggregation when cultured within the PLLA MTAM. At the diameter between 50 and 60 μm, tumor cells could be more evenly distributed thereby resulting in the growth of the tumor cells without exerting massive mitotic pressure to the neighboring tumor cells [33]. The porous walled PLLA MTAM outperformed the solid walled PLLA MTAM because the porosity increased the available pathways for gaseous and nutrients exchange to the level sufficient to support the metabolic activities of the cancer cells [34]. When compared to the cells cultivated within the CellMax fibers, the cell count within the PLLA MTAM were between one to two order higher since MTAM cell culture systems were able to continuously supply nutrients to cells in high density and retained autocrine factors in a closed system [35].

Prior to in vitro and in vivo toxicity test, the IC 50 (half maximal inhibitory concentration of Cisplatin against GFP-GBM on TCP (tissue culture plate) was conducted and the IC 50 was around 15 mM. Based on the results in Figure 3b, regardless of the concentration of Cisplastin, it appeared that upon the administration, the tumor cells cultivated within the PLLA MTAM registered a sharper decrease when compared to the cells cultivated in CellMax systems; indicating that the anticancer drug perfused better into the PLLA MTAM than CellMax fibers which coincided with the presence of the thinner lumen wall of the PLLA MTAM. In the case of in vivo cell cytotoxicity test, it was further confirmed the earlier in vitro results indicating that the PLLA MTAM system were superior to that of the CellMax system, which can be observed through the significantly higher OD (optical density) reading of the negative controls of the tumor cells cultivated within the PLLA MTAM compared to that of the significantly lower OD reading of the negative control of the tumor cells cultivated within the CellMax fibers.

High failure rates are often associated with the lack of reliable preclinical models to predict the effects these newly developed drugs in a setting resembling the complex nature of the tumor environment. One method to overcome this problem is to utilize a patient-derived xenograft model (PDX) [6]. The grafting of cancer cell specimen either subcutaneously or orthotopically in nude mice are thought to be able to be utilized as preclinical models. In order for PDX to be clinically relevant and practical to the point where it is applicable in personalized medicine, the biological fidelity of the transplanted tumors must be maintained when compared to the tumor of origin. Additionally, the time required for such transplantation must be significantly reduced while significantly increasing the overall success of the transplantation. Jonsson et al. reported the drug effects on tumor cells, host animal toxicity and drug pharmacokinetics are the same in animal models using an extended hollow fibers model as those found in traditional xenograft models [7].

Intraperitoneal hollow fiber activity was also found to be a better predictor of xenograft activity when compared to subcutaneous hollow fiber activity or intraperitoneal plus subcutaneous activity combined [8]. Compared to PDX and traditional hollow fiber systems, the PLLA MTAM will benefit patients in terms of relatively quick turn-around time, smaller cell sample needed and of course, a higher drug sensitivity. This will allow oncologist treating a particular patient to know the best anticancer drug that should be administered. Early treatment of cancer will without a doubt increase the survival chances of the patient [9]. MTAM-based drug screening platform can be readily transformed to an effective drug selection protocol for individual treatment. By replacing the cancer cell-line used in current study with patient-derived tumor cell (PDTC), the comprehensive result with the most powerful drug/combinations of drug toward this individual can be identified in a timely fashion.

Additionally, several other limitations were observed in the use of PDX as a standard modality in modeling human cancers. Namely, the loss of tumor microenvironment and immune-response [36], selection for clonal subpopulations that are different from that of the original tumor [37], the diversity in drug metabolism [38] and cost versus effectiveness [39]. Predominately, the grafting of tumors are research tools employed in the pharmaceutical industry with poor response rates observed with repeated lines of traditional chemotherapy, the value of this tool in addressing the continuing challenges in clinical oncology will increase over time.

## 5. Conclusions

The in vitro and in vivo data gathered in this study demonstrated that MTAM is a superior culture substrate for cancer cells when compared to traditional HFs, as well as being a significantly more sensitive and reliable system for anticancer drug screening. The MTAM/HFA system conferred significant benefits in terms of the cost, rapidness, reliability and the reduction in the overall number of required animal used in a study; while maintaining comparable trend outcome compared to the current gold standard-traditional xenograft model. Clinically, we also demonstrated the potential of MTAM/HFA-PDTC, which demonstrated its practicality, ease of use and reliability. In short, the range of benefits of the MTAM/HFA system made it a highly practical phenotypic-based solution that could potentially be applied in applications such as anticancer drug screening for personalized medicine and as a pre-xenograft model the development of anticancer drugs.
